# Draft Genome Sequences of Three Isolates of *Coniothyrium glycines*, Causal Agent of Red Leaf Blotch of Soybean

**DOI:** 10.1128/MRA.00378-19

**Published:** 2019-10-03

**Authors:** Trenna Blagden, Andres Espindola, Kitty Cardwell, Alejandro Ortega-Beltran, Ranajit Bandyopadhyay

**Affiliations:** a Department of Entomology & Plant Pathology, Oklahoma State University, Stillwater, Oklahoma, USA; b National Institute of Microbial Forensics in Food and Agricultural Biosecurity, Oklahoma State University, Stillwater, Oklahoma, USA; c International Institute of Tropical Agriculture, Ibadan, Nigeria; University of California, Riverside

## Abstract

Coniothyrium glycines, the causal agent of red leaf blotch in soybeans, is considered a high-consequence biological agent. With limited genomic information known, there are no molecular genotyping or detection methods available. We report the draft genome sequences of three *C. glycines* isolates, greatly enhancing our knowledge of this species.

## ANNOUNCEMENT

The pycnidial state of a fungal species, Pyrenochaeta glycines, was first suspected of causing red leaf blotch of soybeans in 1955 in Ethiopia (1). Later, the sclerotial state of a fungus causing leaf spot disease was described as Dactuliophora glycines (2). Following extensive phylogenetic analysis, it is now designated Coniothyrium glycines (R. B. Stewart) Verkley & Gruyter (3).

*C. glycines* is found in soybeans in central and southern Africa (1, 4, 5). Previous records indicate that yield losses can reach up to 50% (6). With the United States annually exporting 52.8 million tons of soybeans, a farm cash value of $35.2 billion in 2015 (https://www.ers.usda.gov/topics/crops/soybeans-oil-crops/related-data-statistics/), the threat posed by *C. glycines* could be significant. It is currently unknown if this fungus can survive in the temperate regions of the United States or what the potential damage from its spread to other cultivated legumes would be (7). The availability of genomic sequences would provide valuable knowledge for detection tool development and facilitate our understanding of genetic diversity within *C. glycines*.

*Coniothyrium glycines* 13, 17, and 18 are pycnidial isolates originating from soybean leaves collected by Olalekan Ayinde in September 2016 at ECOWAS Church Farm in Jos Plateau, Nigeria. Isolates grown on potato dextrose agar (PDA) plates were sent to Oklahoma State University (Stillwater, OK), where single hyphal tip isolation was performed. They were subcultured on 10% V8 juice agar plates and maintained at 20°C in darkness. The agar plate surface was scraped into 1 ml of sterile reverse-osmosis (RO) water to collect pycniospores for DNA extraction. The spore solution was transferred into a Precellys 24 VK05 lysing kit 2.0-ml tube with 10 2.7-mm glass beads. The Precellys 24 tissue homogenizer (Bertin Instruments, Montigny-le-Bretonneux, France) run was two 25-second cycles at 6,500 rpm with a 5-second pause between cycles. The genomic DNA was extracted using the DNeasy plant and fungal extraction kit (Qiagen, Germantown, MD). Sequencing of *C. glycines* 13, 17, and 18 was performed using the Nextera kit on an Illumina MiSeq platform and a rapid sequencing kit (SQK-RAD004) with a FLO-MIN106.1 SpotON flow cell (R9.4) for the Oxford Nanopore MinION system. Assembly using a hybrid approach with SPAdes 3.13.0 (8) was conducted for *C. glycines* 17 and with Canu 1.8 (9) for *C. glycines* 13 and 18. The Illumina reads were quality controlled with FastQC 0.11.3, and the MinION reads were quality controlled with Albacore 2.3.4. All relevant sequencing and assembly statistics are summarized in Table 1. BUSCO version 3.0.2 using the BUSCO fungi_odb9 database was used to determine completeness of genome coverage and culture identity confirmed by a BLAST+ 2.8.1 (10) search for a 100% match of the internal transcribed spacer (ITS) region of *C. glycines*. The BUSCO scores for strains 13, 17, and 18 were all above 96% complete genes, and all isolates had the complete ITS region.

**TABLE 1:** Sequencing and assembly statistics of *C. glycines* strain genomes

Isolate	Genome coverage (×)	Total no. of MinION reads used	Illumina avg. read length (bp)	Total no. of Illumina reads used (million)	Genome size (Mbp)	No. of contigs	*N*_50_ (kbp)	G+C content (%)
13	1,722.105	2,020,000	146.39	11.9	32.75	260	520	52.19
17	1,697.311	518,282	142.63	13.7	36.88	309	269	52.19
18	1,691.057	97,848	146.13	10.8	33.10	262	436	52.19

### Data availability.

This whole-genome shotgun project has been deposited at DDBJ/ENA/GenBank under the accession numbers SPUQ00000000 (strain 18), SPUR00000000 (strain 17), and SPUS00000000 (strain 13). The versions described in this paper are versions SPUQ02000000, SPUR01000000, and SPUS02000000. The raw sequence data were deposited under SRA accession numbers SRR8655434 to SRR8655442.
